# Influence of Low-Pressure Treatment on the Morphological and Compositional Stability of Microscopic Ettringite

**DOI:** 10.3390/ma14112720

**Published:** 2021-05-21

**Authors:** Patrick A. Kißling, Franziska Lübkemann, Tabea von Bronk, Dario Cotardo, Lei Lei, Armin Feldhoff, Ludger Lohaus, Michael Haist, Nadja C. Bigall

**Affiliations:** 1 Institute of Physical Chemistry and Electrochemistry, Leibniz Universität Hannover, 30167 Hanover, Germany; patrick.kissling@pci.uni-hannover.de (P.A.K.); franziska.luebkemann@pci.uni-hannover.de (F.L.); armin.feldhoff@pci.uni-hannover.de (A.F.); 2 Institute of Building Materials Science, Leibniz Universität Hannover, 30167 Hanover, Germany; t.von-bronk@baustoff.uni-hannover.de (T.v.B.); d.cotardo@baustoff.uni-hannover.de (D.C.); lohaus@baustoff.uni-hannover.de (L.L.); haist@baustoff.uni-hannover.de (M.H.); 3 Chair for Construction Chemistry, Technische Universität München, 85747 Munich, Germany; lei.lei@bauchemie.ch.tum.de

**Keywords:** morphology, chemical composition, ettringite, low-pressure, Pawley fit

## Abstract

The impact of low-pressure treatment on the crystal structure, morphology, and chemical composition of ettringite, due to their major importance with respect to processability (i.a., drying conditions) and to the analysis of ettringite-containing samples, is examined utilizing X-ray diffraction, thermogravimetric analysis, Raman spectroscopy, and environmental scanning electron microscopy. Synthetic ettringite was treated for various durations (5 min up to 72 h) and at two different levels of low-pressure (4.0 mbar and 60 µbar). Evaluation showed a correlation between the procedural parameters (time and pressure), the chemical composition, and the morphology of ettringite. The experiments reveal that, when exposed to 4 mbar pressure, nearly no changes occur in the ettringite’s morphology, whereas the crystals undergo swelling and slight deformations at very low pressures (60 µbar and 35.3 nbar), which is attributed to the loss of bound water and the partial transformation from ettringite to quicklime, anhydrite, and calcium aluminate. Furthermore, the strongly dehydrated ettringite shows the same morphology.

## 1. Introduction

Ettringite (Ca_6_Al_2_(SO_4_)_3_(OH)_12_·26H_2_O) is formed as small needle-shaped crystals on the surface of cement particles during the early stages of hydration [1,2,3,4,5,6] and thus is of significant technical importance, as it influences the rheological properties of fresh cement suspensions. Furthermore, ettringite is used as white pigment in paper production [7,8] or for high-coverage emulsion wall paints [1]. Previous works proved that the crystal structure of ettringite consists of dodecahedrally coordinated calcium ions and octahedrally coordinated aluminium ions forming face-linked hexagonal prisms along the c-axis [1,9,10,11] (Figure S1). The pioneering works of Skoblinskaya et al. revealed prominent changes in the crystal structure: a shrinkage in the cell parameters at different hydration stages using isobar treatment at 8.0 µbar in a range of 25 °C to 325 °C was observed [5,6]. Within their study, four different types of water (adsorption sites) were identified in the ettringite structure [5,6]. Additionally, characterizations of the changes in ettringite’s crystal structure were performed after the treatment at ambient pressure in a range of 25 °C to 200 °C for up to 7 h [12]. The above-mentioned works have already demonstrated that the bound crystal water of ettringite can be effectively removed by low-pressure or elevated temperatures. Nevertheless, a set of parameters where the original crystal structure of ettringite can be preserved upon the removal of adsorbed water is still sought after. To the best of our knowledge, no systematic study has investigated the impact of different levels (4 mbar, 60 µbar, and 35.3 nbar) of low-pressure yet. Due to widely used characterization techniques and processing conditions, as well as the investigation of growth kinetics, the stability of the crystal structure and morphology under low-pressure treatment is of paramount importance. In particular, high performance imaging techniques, such as transmission electron microscopy (TEM) or scanning electron microscopy (SEM), require high-vacuum treatment (0.9 nbar or 35.3 nbar). This, in turn, raises the question of which techniques are suitable for the noninvasive and nondestructive analysis of ettringite’s crystallinity, chemical composition, and morphology, which are relevant, e.g., for the properties of cementitious suspensions [3,13,14,15,16,17,18].

This article documents a study of the chemical composition, crystallinity, and morphology of synthetic ettringite depending on the level (4 mbar, 60 µbar, and 35.3 nbar) and the duration (up to 72 h) of low-pressure treatment to which the sample was exposed. X-ray diffraction analysis (XRD) was used to gain insight into the stability of the crystals under the various pressure conditions, while the cell parameters were investigated by employing the Pawley fit method [19]. The bound water content was evaluated by thermogravimetric analysis (TGA) as well as Raman spectroscopy (Raman) to distinguish between hydroxide groups and bound water while giving insight into the chemical composition. Scanning transmission electron microscopy coupled with energy-dispersive X-ray spectroscopy (STEM-EDXS) was used to clarify the chemical composition of the fully dehydrated sample. To identify the degree of crystallinity of the strongly dehydrated ettringite, selected area electron diffraction (SAED) was employed. The morphology of ettringite was investigated by environmental scanning electron microscopy (ESEM). Through time-dependent measurements with the characterization techniques mentioned above, the chemical, crystallographic, and morphological stability of ettringite at various levels of low-pressure was examined.

## 2. Materials and Methods

### 2.1. Materials

The main materials for this study were CASUL^®^ H1i from Remondis, aluminium sulphate octadecahydrate (≥98%) from Carl Roth, calcium hydroxide (≥95%) from Sigma-Aldrich, Millipore water (18.2 MΩ·cm) cleaned by an Arium 611DI from Sartorius GmbH & Co. KG, Goettingen, Germany (in the following referred to as water), and liquid nitrogen (≥99.999%) from Linde. As an internal standard for XRD measurements, silicon (97.5%) from Riedel-de Haën was used. The synthetic ettringite was stored at inert conditions (25 °C, 1.015 bar, Ar atmosphere) for the whole study.

### 2.2. Methods

#### 2.2.1. Synthesis of Ettringite

Following Struble et al. [20], a solution of aluminium sulphate octadecahydrate [Al_2_(SO_4_)_3_·18H_2_O, 38.3 mM, 1 Veq.] and a saturated solution of calcium hydroxide [Ca(OH)_2_, 10 Veq.] in water were prepared. The alkaline solution was filtrated using a Buchner funnel with a glass fibre filter (retention 0.4 µm, d = 125 mm; MN-GF 5 by Macherey-Nagel GmbH, Co. KG, Dueren, Germany). Both solutions were mixed quickly (30 s) under vigorous stirring (500 rpm, Hei-Torque Precision 400 by Heidolph Instruments GmbH, Co. KG; anchor AR 19 PTFE, d_shaft_ = 8 mm). Instantaneous precipitation of ettringite was evident from the solution turning turbid. After centrifugation (60 min, 10,000 G, Sigma 3-18KS, 20 °C), the precipitate was decanted. To remove the adsorbed water, or rather remaining reaction medium, with neither phase transition (25 °C, 1.015 bar) nor the formation of lime [CaCO_3_] (Ar atmosphere), the precipitate was dried in a vacuum furnace (Vakuumschrank VO29 by Memmert GmbH + Co. KG, Büchenbach, Germany) for 24 h at the aforementioned ambient and inert conditions.

#### 2.2.2. Influence of Low Pressure

To assess the influence of low-pressure on the morphology and chemical composition of ettringite, various durations (5 min up to 72 h) and levels of pressure (4.0 mbar, 60 µbar, and 2.8 nbar) were applied. These levels of pressure were chosen as 4.0 mbar, which is near the triple point of water (6.1 mbar) [21,22], 60 µbar, the standard pressure of freeze-drying devices, and 2.8 nbar, a common pressure during scanning electron microscopy. For achieving different pressure values (4 mbar and 60 mbar), a freeze-dryer (Alpha 1-2 LDplus by Martin Christ Gefriertrocknungsanlagen GmbH) coupled with a two-stage rotary vane pump (RV 3F by Edwards Vacuum) was used. A high vacuum (2.8 nbar) was realized in the antechamber of the scanning electron microscope Zeiss Supra VP 55. Each treatment was performed on a different sample taken out of the same batch.

#### 2.2.3. X-ray Diffraction and Pawley fit

The crystallinity was investigated by X-ray diffraction (XRD) using a Bruker D8 Advance in reflection mode. It was operated at 20 °C, 40 kV, and 40 mA using Cu-K_α_ radiation. Each measurement was done 30 min after the low-pressure treatment in a 2θ range from 5° to 70° with a step size of 0.006571365° and 4 s per step, resulting in a total measurement time of 7.25 h. The powder of each sample was transferred into an X-ray amorphous PVC powder carrier and smoothed to ensure no sample displacement. To guarantee that the shift of the reflections in the XRD was not caused by a sample displacement error in the preparation, 2.2 wt% (11 mg) silicon was added as an internal standard. The diffraction patterns were evaluated by the database of the Powder Diffraction File (PDF-2) 2020 of the International Centre for Diffraction Data (ICDD).

Furthermore, the cell parameters were analysed with a Pawley fit [19] using the data provided by Hartman et al. [23], the measured XRD data of this work, and the software TOPASv6 by Bruker.

#### 2.2.4. Thermogravimetric Analysis

To evaluate the content of bound water, which accounts for 46% of the molar mass of ettringite and is responsible for the stability of its crystal structure, any synthesized or dried sample was investigated by thermogravimetric analysis (TGA) using a TGA/DSC 3+ from Mettler-Toledo GmbH. The samples were transferred into aluminium oxide crucibles (70 µL) before measurement. It was operated in a temperature range from 20 °C to 1100 °C under a nitrogen flow of 25 mL/min and a heating ramp of 5 °C/min, followed by holding the temperature at 1100 °C for 15 min. The data was normalized by mass (mg), derived once by time (s), multiplied by 3600 (for plotting in h), and plotted against temperature (°C).

#### 2.2.5. Quick Assessment of Mass Loss

For an assessment of mass loss of ettringite after the treatment for various durations (5 min up to 72 h) and different levels of pressure (4.0 mbar and 60 µbar), a balance Practum224-1S from Sartorius GmbH & Co. KG was used before and after treatment (Figures S2 and S3, Tables S1 and S2).

#### 2.2.6. Environmental Scanning Electron Microscopy

To reassess the results about the morphology of ettringite derived from the Pawley fit, environmental scanning electron microscopy (ESEM) using a Zeiss Supra VP 55 equipped with a cold field emission gun and a 4-quadrant backscatter electron detector (QBSD) was implemented as an imaging method. The acceleration voltage was 10 kV, the current 6 nA, and variable pressure 1.0 mbar and 60 µbar for each measurement. The powder was transferred onto an adhesive carbon disk and was cleaned of excess sample with a compressed air gun. Each sample was degassed in the antechamber for 2.5 min for safety reasons. At a pressure of 375 nbar in the antechamber, the “variable pressure” mode was started. For the investigation of the influence of the electron beam during the micrograph acquisition on the morphology, the treatment at 0.5 mbar and 10,000 times magnification was recorded for 2.2 min (Section 3.2.3).

#### 2.2.7. Scanning Electron Microscopy

For the scanning electron microscopy (SEM) (Carl Zeiss AG, Oberkochen, Baden-Wurttemberg, Germany), a Zeiss Supra VP 55 equipped with a cold field emission gun and a 4-quadrant backscatter electron detector (QBSD) was used as an imaging method. The acceleration voltage was 10 kV, the current 6 nA, and the pressure 375 nbar for each measurement. The powder was transferred on an adhesive carbon disk and was cleaned of excess sample with a compressed air gun. Each sample was degassed in the antechamber for 2.5 min for safety reasons.

#### 2.2.8. Raman Spectroscopy

To distinguish the bound water and hydroxide in the crystal structure of ettringite, further structural analysis was performed by Raman spectroscopy (Bruker Corporation, Billerica, MA, USA) using a Bruker Senterra Raman spectrometer equipped with an Olympus LMPIanFl N 50x lens with the FlexFocus^TM^ system for confocal depth profiling and an ANDOR DU420-OE with a thermoelectric cooling system as a charge-coupled device (CCD). The green laser (λ = 532 nm) was operated with a total power of 20 mW and a spectral resolution of 3 cm^−1^ to 5 cm^−1^. Each spectra was collected with an acquisition time of 5 s per scan, merging five consecutive scans in a range of 50 cm^−1^ to 1555 cm^−1^, 1522 cm^−1^ to 2739 cm^−1^, and 2705 cm^−1^ to 3705 cm^−1^. The grating calibration was controlled by checking the position of the Raman line of a Si standard at 519.9 cm^−1^ [24]. The samples were transferred as powder on a glass slide.

#### 2.2.9. Transmission Electron Microscopy and Energy-Dispersive X-ray Spectroscopy

The local distribution of elements before and after the treatment of ettringite at 60 µbar for 48 h was investigated by scanning transmission electron microscopy (JEOL Ltd., Tokyo, Japan) coupled with energy-dispersive X-ray spectroscopy (STEM-EDXS) using a JEOL JEM-2100F-UHR (C_s_ = 0.5 mm and C_C_ = 0.5 mm) equipped with a field-emission gun. The acceleration voltage was 200 kV and the pressure 0.9 nbar for each measurement. Prior to EDXS, micrographs were taken in high angle annular dark-field (HAADF) scanning transmission electron (STEM) mode. The powder was transferred on a carbon coated copper TEM grid.

#### 2.2.10. Selected Area Electron Diffraction

The zone axis pattern before and after the treatment of ettringite at 60 µbar for 48 h was investigated by selected area electron diffraction (SAED) (JEOL Ltd., Tokyo, Japan) using a JEOL JEM-2100F-UHR (C_s_ = 0.5 mm and C_C_ = 0.5 mm) equipped with a field-emission gun. The acceleration voltage was 200 kV and pressure 0.9 nbar for each measurement, and all samples were transferred as powder on a carbon coated copper TEM grid.

## 3. Results and Discussion

### 3.1. Synthetic Ettringite

**Figure 1:**
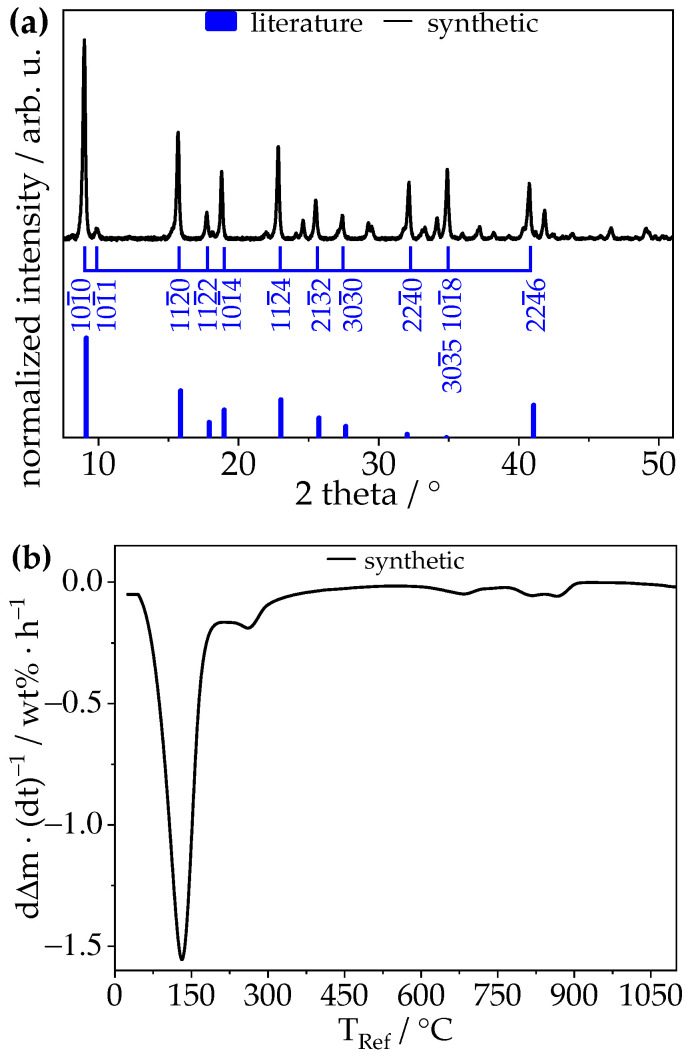
(**a**) Powder X-ray diffraction pattern of synthetic ettringite (black) and its literature data reflections (blue) [9,23] indexed using Bravais–Miller indices according to a trigonal symmetry in a hexagonal cell and (**b**) TGA data plotted as mass normalized time derived weight loss (dΔm·(dt)^−1^) per hour against the reference temperature of synthetic ettringite with a heating rate of 5 °C/min.

The synthetic colourless powder was analysed using XRD and TGA and thus was proven to be ettringite [Ca_6_Al_2_(SO_4_)_3_(OH)_12_·26H_2_O]. Figure 1a shows the diffractogram comparing the measured 2θ data with its most prominent reflections (I_reflection_ > 0.1 I_max_) in the angle range of 7.5° ≤ 2θ ≤ 51° [23], which align with the data by Hartman et al. [23]. Both discrepancies in the intensity of the reflections at 2θ = 32.03° and 2θ = 34.84° compared with the literature are most likely induced by the faceting of the crystals. This is especially prominent for the reflection at 2θ = 34.84°, due to two different underlying crystal planes (($10\bar{1}8$) and ($30\bar{3}5$)) [23], which are given by their Bravais–Miller indices according to a trigonal symmetry in a hexagonal cell. In the TGA measurement depicted in Figure 1b, a large mass loss of 34.62% can be seen between 50 °C and 210 °C. This is attributed to the decomposition of ettringite to calcium aluminate monosulphate [AFm-14H; Ca_4_Al_2_(SO_4_)_3_(OH)_12_·8H_2_O] and basanite [CaSO_4_·0.5H_2_O], as described by Hall et al. [25], see Equation (1):

Ca_6_Al_2_(SO_4_)_3_(OH)_12_·26H_2_O → Ca_4_Al_2_(SO_4_)_3_(OH)_12_·8H_2_O + 2CaSO_4_·0.5H_2_O + 17H_2_O (1)

Further dehydration of basanite at 225 °C ± 25 °C and dehydration of AFm-14H at 775 °C ± 125 °C are visible. At 900 °C ettringite is fully decomposed to quicklime [CaO], anhydrite [CaSO_4_], and calcium aluminate [(CaO)_x_(Al_2_O_3_)_y_] [26,27,28]. The total mass loss of 46.41% matches with the bound water content of ettringite.

**Figure 2:**
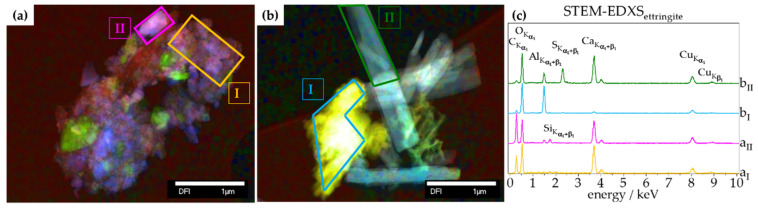
STEM micrographs of pristine ettringite (**a**,**b**) sites of EDXS and (**c**) corresponding STEM-EDXS spectra of different sites.

In addition, STEM-EDXS measurements were recorded from different sites to analyse the chemical composition of the pristine ettringite. The STEM micrographs showed a needle-like shape of the as-synthesized ettringite (Figure 2a) and residues of the unreacted precursor [Al_2_(SO_4_)_3_·18H_2_O and Ca(OH)_2_] (Figure 2b). The corresponding STEM-EDXS analysis showed an elemental distribution and elemental stoichiometric ratio indicating ettringite and residues of unreacted precursor (Figure 2).

### 3.2. Treatment of Ettringite with Low-Pressure

The synthetic ettringite was exposed to different low-pressure values, as described in Section 2.2. To analyse the influence of low pressure on the crystal structure, morphology, and chemical composition of ettringite, three different aspects are considered. First, the chemical composition of ettringite after different durations and levels of exposure to low-pressure is characterized by TGA, XRD, Raman, and STEM-EDXS (Section 3.2.1). Second, changes of cell parameters are assessed by SAED and the Pawley fit of the measured XRD data (Section 3.2.2). Third, the ettringite’s crystal morphology is analysed by SEM and ESEM (Section 3.2.3).

#### 3.2.1. Chemical Composition—TGA, XRD, Raman, STEM-EDXS

**Figure 3:**
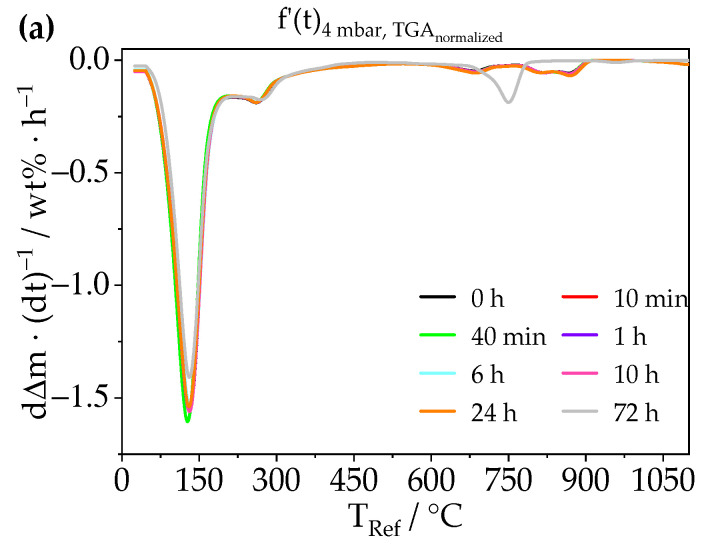

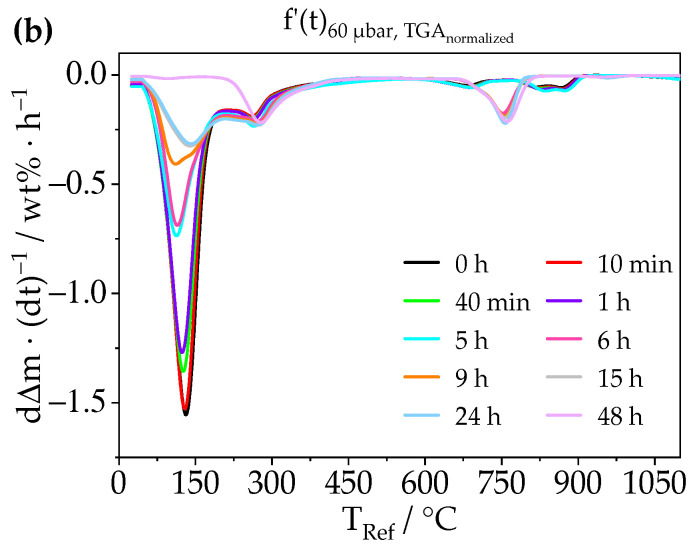
TGA data plotted as mass normalized time derived weight loss (dΔm·(dt)^−1^) per hour against the reference temperature of synthetic ettringite with a heating rate of 5 °C/min after treatment at two different levels of low pressure (**a**) 4 mbar and (**b**) 60 µbar for various durations (up to 72 h); full time frame depicted in Figure S4.

The depletion of the bound water induced by different pressure treatments was investigated by TGA measurements (Figure 3). The change of chemical composition by depletion of water is shown by the decrease of the first peak at 130 °C ± 80 °C, which correlates to the main decomposition of ettringite (Equation (1)). At 4 mbar, the main decomposition peak shrinks almost linear over 72 h by an amount of 9.4%. At 60 µbar, the same degree of alteration is already reached after 30 min (Figures S4 and S5, and Table S3). At both stages the theoretical ettringite composition should have changed from Ca_6_Al_2_(SO_4_)_3_(OH)_12_·26H_2_O to Ca_6_Al_2_(SO_4_)_3_(OH)_10.8_·23.4H_2_O. Following from this, lower pressure seems to destroy ettringite more quickly. It is also remarkable that the range of the complete dehydration of AFm-14H at 775 °C ± 125 °C becomes a single peak at 730 °C ± 60 °C, and both ranges have the same integral. This change appears after 72 h at 4 mbar and already after 6 h at 60 µbar. Furthermore, at 60 µbar, the decrease of ettringite’s main decomposition peak is not linear but rather follows an exponential decay until 36 h (Figure S5 and Table S3). In the last 12 h of the treatment with low pressure, the remaining 20% of ettringite is destroyed, since the main decomposition peak at 130 °C ± 80 °C disappears.

**Figure 4:**
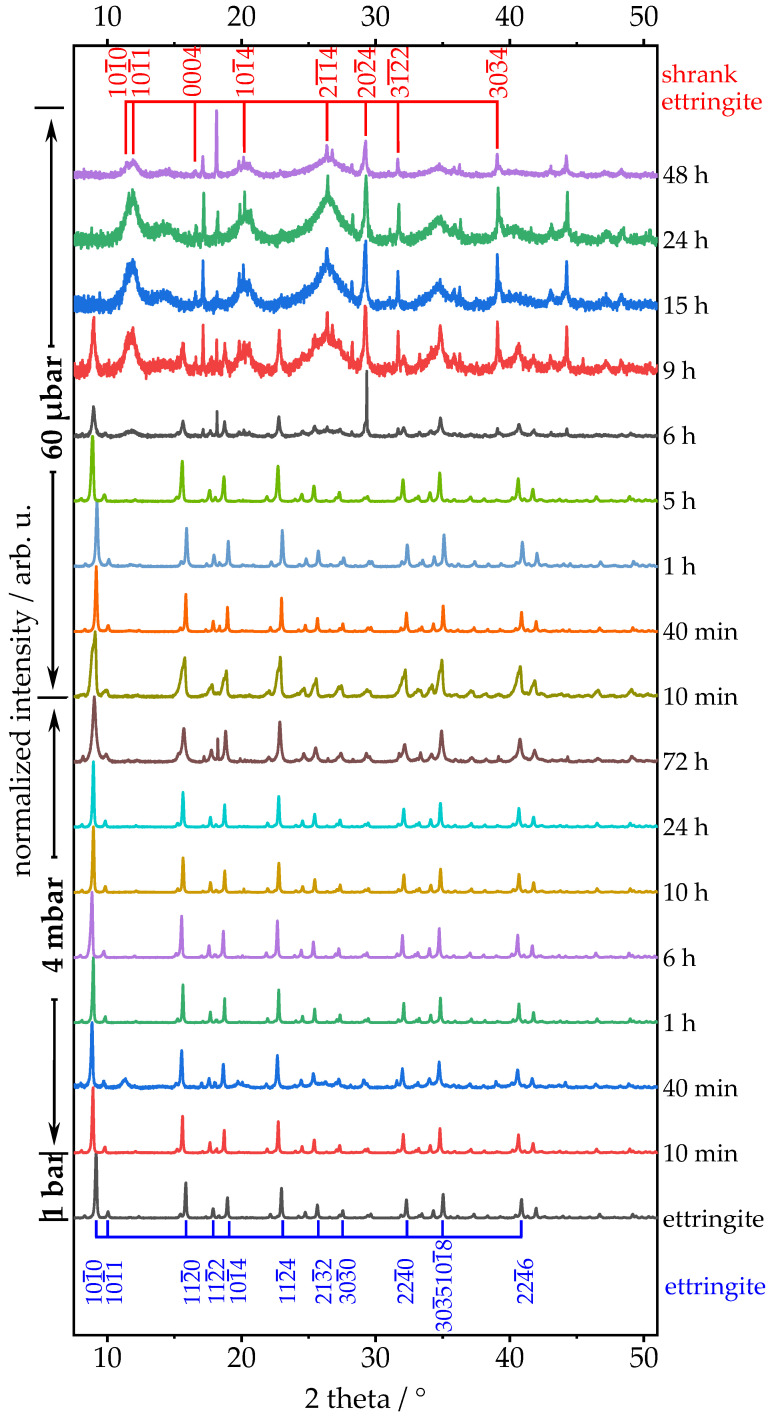
Identification of ettringite reflections (blue) and shrunk ettringite (red) in the diffraction pattern of synthetic ettringite; after treatment at two different levels of low pressure (4 mbar and 60 µbar) for various durations (up to 72 h), each treatment was performed on a different sample from the same batch. Bravais–Miller indices are given according to a trigonal symmetry in a hexagonal cell; full time frame depicted in Figure S6.

Many temperature studies suggested that the dehydration of ettringite leads to a change of its crystal structure [5,6,29,30,31]. Treatment with different levels of low-pressure for various durations changes the chemical compositions as well as the characteristic XRD pattern of ettringite. The XRD patterns of low-pressure treated samples are shown in Figure 4. At 4 mbar, the reflection $10\bar{1}0$, originating mainly from bound water, especially begins to broaden [5,6,12,29,30,31]. This behaviour can be caused either by shrinking crystallite sizes or by defects in the crystal structure and, in our case, is most probably attributable to dehydration. At the lower pressure of 60 µbar, the broadened reflections lose intensity over time until after 9 h, the intensity of reflection $10\bar{1}0$ is equal to reflection $10\bar{1}2$, with an intensity of 2.5% relative to pristine ettringite. After 12 h, reflection $10\bar{1}0$ is ultimately eliminated. Over the following 36 h, nearly all reflections are diminished, as either the structure of ettringite is destroyed or the remaining ettringite becomes X-ray amorphous [5,6,12].

When comparing TGA and XRD results, there seems to be an interdependency between the aforementioned decomposition peak in the TGA at 730 °C ± 60 °C and the reflection in the XRD at 29° after 6 h at 60 µbar (Figure 4). The corresponding phase is indexed as CaCO_3_, which forms over time due to carbon dioxide in the air [12,32,33], see Equation (2):

CaO + CO_2_ → CaCO_3_ (2)

**Figure 5:**
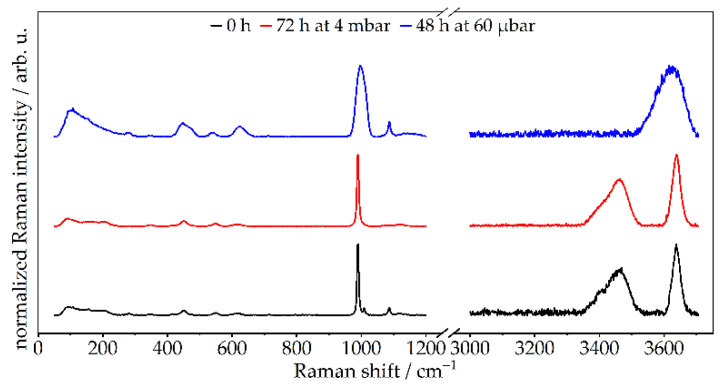
Raman spectra of pristine synthetic ettringite and treated ettringite after treatment at two different levels of low pressure (4 mbar for 72 h and 60 µbar for 48 h).

To identify whether the removal of water delineated by TGA and XRD was induced by the removal of hydroxide groups or bound water, Raman was used as a complementary structural method. This technique shows that the stretching vibrations of the hydroxide groups (3644.0 cm^−1^ ± 42.0 cm^−1^) are preserved in both cases, but the stretching bands of the bound water (3444.8 cm^−1^ ± 102.8 cm^−1^) [24,34] disappear after 48 h at 60 µbar (Figure 5). Hence, it is clear that the bound water of ettringite was fully extracted after 48 h at 60 µbar. The full assignment of the characteristic bands of vibrations of pristine ettringite and of the treated samples is shown in Table S4.

**Figure 6:**
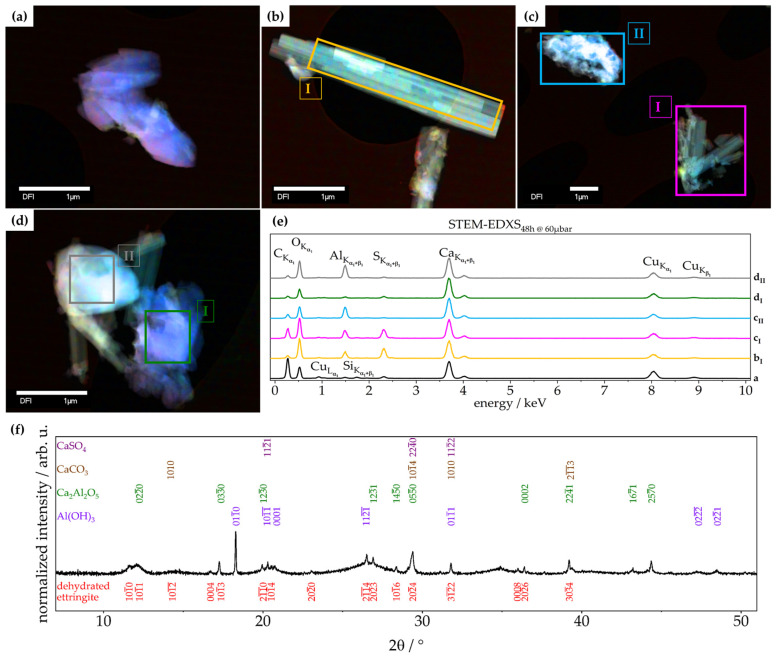
STEM micrograph of ettringite treated at 60 µbar for 48 h; (**a**–**d**) sites of EDXS and (**e**) STEM-EDXS spectra of different sites; (**f**) X-ray diffractogram showing identified phases of retrogressed ettringite after treatment at 60 µbar for 48 h; CaCO_3_ [35], Ca_2_Al_2_O_5_ [36], Al(OH)_3_ [37], and CaSO_4_ [38].

**Figure 7:**
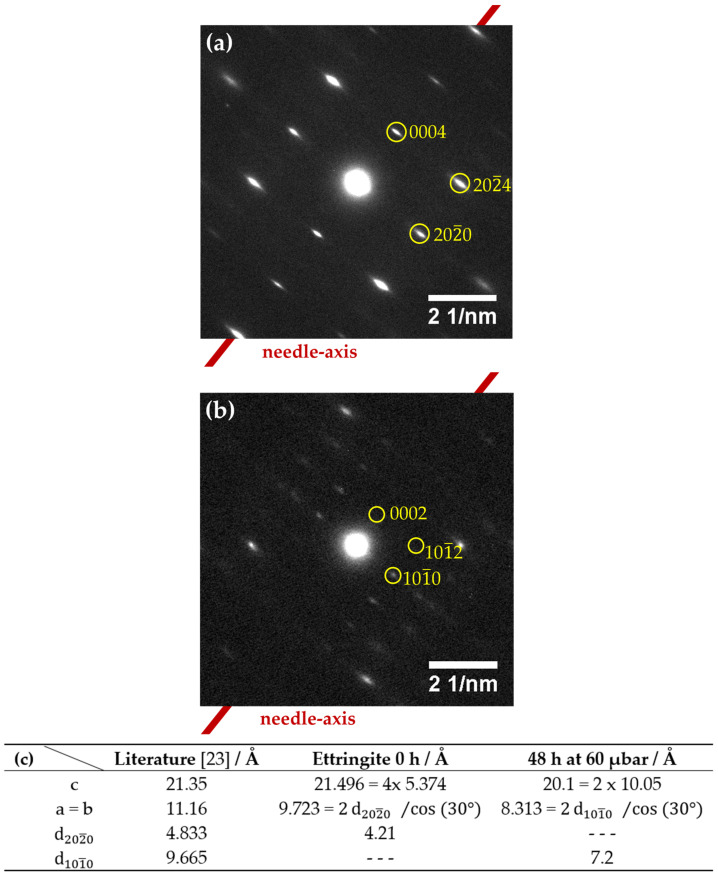
SAED micrographs of ettringite; 200 kV, 50,000 times magnification; (**a**) 550 nm selected circular area on pristine ettringite; (**b**) 250 nm selected circular area on dehydrated ettringite after a treatment of 48 h at 60 µbar; [$10\bar{1}0$] zone axis; Bravais–Miller indices of both patterns are indexed according to a hexagonal unit cell; and (**c**) cell parameters derived from TEM-based SAED of pristine ettringite and ettringite treated at 60 µbar for 48 h compared to data by Hartman et al. [23].

To link the XRD of fully dehydrated ettringite (after treatment at 60 µbar for 48 h) with the results of TGA and Raman, scanning transmission electron microscopy coupled with energy-dispersive X-ray spectroscopy (STEM-EDXS) was employed. This performed high-vacuum treatment (0.9 nbar) had no effect on the morphology, as the sample was already fully dehydrated. Combining these results with the elemental distribution and elemental stoichiometric ratio derived from STEM-EDXS, the following compounds could be indicated as the most probable dehydration products of ettringite: CaCO_3_, Al_2_O_3_, CaSO_4_, CaO, (CaO)_x_(Al_2_O_3_)_y_. Apart from Al_2_O_3_, all of these phases are found in the XRD of retrogressed ettringite (Figure 6f). The unidentified reflections did not match with reflections from the PDF-2 2020 database of ICDD. Based on the needle-like shape (Figure 6b) and an elemental distribution similar to pristine ettringite (Figure 2), as well as the fact that after dehydration, the zone axes were found to be exactly half compared to pristine ettringite, it is assumed that the unidentified reflections in XRD belong to ettringite with different cell parameters (Figure 7).

In summary, TGA showed the expected decomposition of ettringite (Equation (1)) as well as a correlation between bound water content and vacuum treatment conditions. Furthermore, Raman spectroscopy showed that the bound water of ettringite is extracted first. By applying 4 mbar, ettringite loses linearly up to 6.6 wt% of bound water after 72 h of treatment. By a lower pressure of 60 µbar, this extraction is faster, as after 20 min, 5.8 wt% of bound water is extracted, and follows an exponential decay. XRD analysis also shows a retrogression of ettringite’s diffraction pattern, wherein the process for 4 mbar is barely visible compared to 60 µbar being distinctive. STEM-EDXS analysis confirmed ettringite’s chemical composition and needle-like shape (Figure 2). It was respectively shown that, as indicated by TGA and XRD, CaCO_3_ is formed over time during the treatment of ettringite with low-pressure (Figure 2a). Furthermore, this technique indicates that, aligning with the known phases after a complete decomposition of ettringite [26,27,28], CaCO_3_, Al_2_O_3_, CaSO_4_, CaO, and (CaO)_x_(Al_2_O_3_)_y_ are the most probable retrogressed phases of ettringite.

#### 3.2.2. Crystallographic Assessment—SAED, Pawley fit

TEM-based selected area electron diffraction (SAED) of pristine ettringite and retrogressed ettringite after a treatment of 48 h at 60 µbar was employed. This technique proves the crystallinity in both cases but reveals differences in the [$10\bar{1}0$] zone axis pattern (Figure 7). In the TEM vacuum (0.9 nbar), pristine ettringite has become elongated by about 0.7% along the c-axis (1.007 c_0_) but shrinks at about 14.7% in directions perpendicular to the c-axis (0.853 a_0_). This elongation of the reflection spots is perpendicular to the c-axis—in this case, also the needle-axis—which hints at disorder in this direction, due to partial dehydration under these harsh conditions (Figure 7a). The strongly dehydrated ettringite (Figure 7b) shrank by about 6.2% along the c-axis (0.938 c_0_) and by about 25.5% in directions perpendicular to the c-axis (0.745 a_0_), relative to the results shown by Hartman et al. [23]. This pronounced shrinkage seems reasonable and has been reported by Skoblinskaya et al. [5,6].

The fact that after dehydration the needle-like shape of ettringite is retained (Figure 6b), as well as the zone axes found to be exactly half compared to pristine ettringite, leads to the assumption that the unidentified reflections in XRD belong to ettringite with different cell parameters (Figure 7).

**Figure 8:**
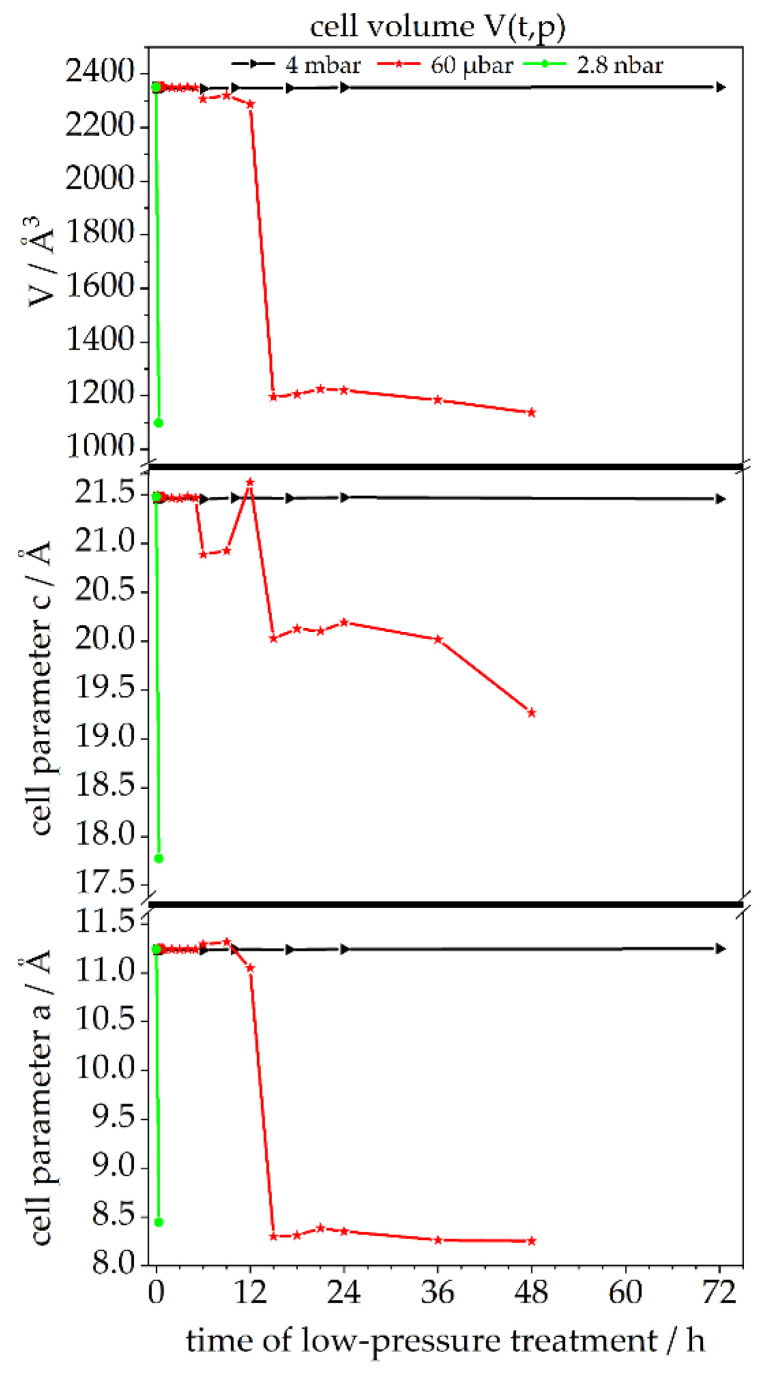
Cell parameters a and c and cell volume V derived from Pawley fits of powder XRD data; retrogression of the cell of synthetic ettringite after treatment at three different levels of low pressure (4 mbar: black; 60 µbar: red; 2.8 nbar: green) for various durations (up to 72 h).

The changes to crystal structure as described in the section above are further investigated by applying the Pawley fit [19] on the diffraction patterns of ettringite after treatment at two different levels of low pressure (4 mbar and 60 µbar) for various durations (up to 72 h). The Pawley fit was chosen to assess changes to ettringite’s cell parameters, as there is no model to describe the occupation of lattice sites of the bound water molecules at the different stages of the dehydration process of ettringite, which is essential for Rietveld refinement. For pristine ettringite, the cell parameters a_0_ = 11.24 Å, c_0_ = 21.48 Å and volume V_0_ = 2351 Å³ are in perfect accordance with the literature [5,6,9,23]. At 4 mbar, cell parameter a remains at 11.24 Å ± 0.004 Å (0.9998 a_0_ ± 0.0003 a_0_), and cell parameter c remains at 21.46 Å ± 0.007 Å (0.9993 c_0_ ± 0.0003 c_0_) over the whole treatment period of 72 h (Figure 8 and Table S5). Although the reported shrinkage of cell parameter c is different, the calculated data show a good accordance to Shimada et al. [12].

The treatment of ettringite at 60 µbar exposes different stages. For the first 5 h, cell parameter a remains at 11.24 Å ± 0.004 Å (1.0001 a_0_ ± 0.0004 a_0_), and cell parameter c remains at 21.47 Å ± 0.01 Å (0.9998 c_0_ ± 0.0004 c_0_). Additional treatment for 4 h (total 9 h at 60 µbar) leads to an expansion of cell parameter a to 11.30 Å ± 0.01 Å (1.006 a_0_ ± 0.0015 a_0_) and a shrinkage of cell parameter c to 20.91 Å ± 0.03 Å (0.9734 c_0_ ± 0.0012 c_0_). After 12 h of treatment, both cell parameters show shrinkage to cell parameter a = 11.05 Å (0.983 a_0_) and cell parameter c = 20.63 Å (0.96 c_0_). The last dehydration step seems to occur between 12 h and 15 h. Afterwards, cell parameter a = 8.31 Å ± 0.05 Å (0.74 a_0_ ± 0.01 a_0_) and cell parameter c_0_ = 19.96 Å ± 0.34 Å (0.93 c_0_ ± 0.02 c_0_) remain stable for at least 33 h (total 48 h at 60 µbar). A correlation between the maximum retrogressed cell parameters of ettringite’s crystal structure, TGA, and Raman spectroscopy is depicted in Figure S7. The sensitivity of ettringite to very low-pressure conditions is investigated via scanning electron microscopy (SEM) operating at a pressure of 2.8 nbar, which, according to previous investigation, should lead to a very strong dehydration and fast transformation. A treatment of 20 min in the antechamber is, according to the Pawley fit, sufficient to deform the original crystal structure of ettringite, which can be derived from Figure 8 and Table S6. The deformation of the needle-like shape particles within the SEM measurement is shown in Figure S8. Hence, a condition for the imaging analysis of the morphology of ettringite is a working pressure as high as possible. Therefore, environmental SEM (ESEM) is an adequate imaging method, as the working pressure is 1.0 mbar. Micrographs obtained by ESEM at 1.0 mbar are not as high resolution as normal SEM micrographs achieved in a high vacuum but can depict the morphology of ettringite without changing the chemical composition due to low pressure.

#### 3.2.3. Morphology—SEM, ESEM

**Figure 9:**
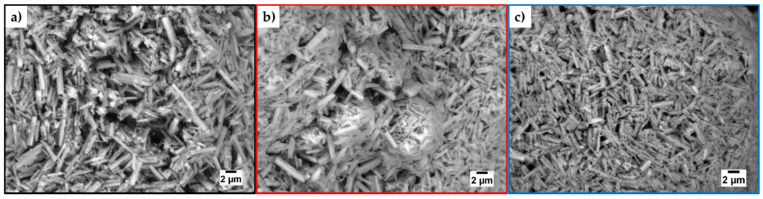

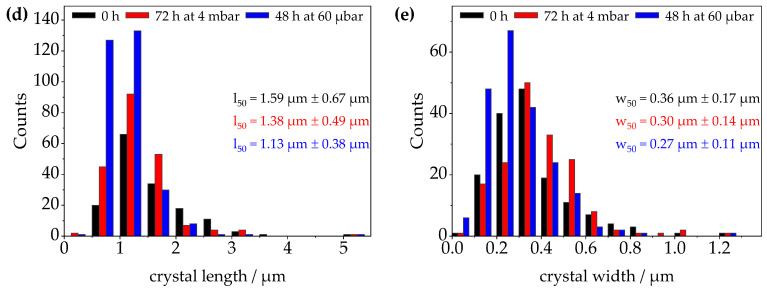
ESEM micrographs of (**a**) pristine synthetic ettringite and treated ettringite after treatment at three different levels of low pressure, (**b**) 4 mbar for 72 h, (**c**) 60 µbar for 48 h, (**d**) respective length, and (**e**) width of ettringite’s crystals derived from ESEM micrographs.

After treatment at two different levels of low pressure (4 mbar and 60 µbar) for various duration (72 h and 48 h), the ettringite needle-shaped particles were investigated via environmental scanning electron microscopy (ESEM) (Figure 9a–c). Pristine ettringite crystals have a mean length of l_50_ = 1.59 µm ± 0.67 µm, a mean width of w_50_ = 0.36 µm ± 0.17 µm, and an aspect ratio of AR = 6.6 ± 4.9. These values shift to a more homogenous distribution after 72 h at 4 mbar with l_50_ = 1.38 µm ± 0.49 µm, w_50_ = 0.30 µm ± 0.14 µm, and AR = 7.0 ± 5.0. By changing the parameters to 48 h at 60 µbar, the crystals shrunk to l_50_ = 1.13 µm ± 0.38 µm, w_50_ = 0.27 µm ± 0.11 µm, and AR = 5.6 ± 3.6 (Figure 9d,e).

**Figure 10:**
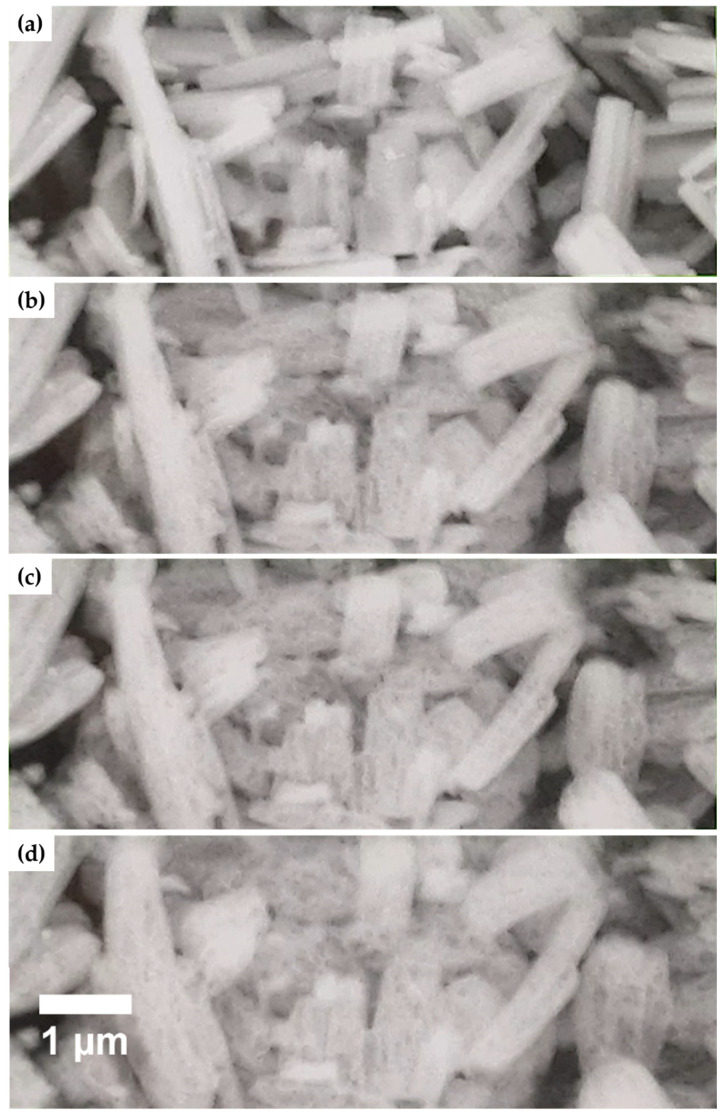
ESEM micrographs of the recorded decomposition of pristine ettringite’s crystals by the electron beam over time; 1 mbar, 10 kV, 6 nA, 10,000 times magnification, 4 s per scan; (**a**) 1 scan, (**b**) 12 scans, (**c**) 23 scans, and (**d**) 33 scans.

To exclude the influence of the electron beam of the ESEM during the micrograph’s acquisition on the morphology, treatment at 0.5 mbar and 10,000 times magnification was recorded (Figure 10). It is clearly visible that the ettringite crystals swell after 12 scans (equivalent to 48 s in the electron beam). After 17 scans (equivalent to 76 s in the electron beam), the first cavities are formed by the explosive evaporation of the bound water of ettringite. After a total of 33 scans (equivalent to 136 s in the electron beam), no new cavities can be observed. Furthermore, the crystals’ length shrinks only slightly, while their width increases considerably, which must be taken into account for future morphological investigations implementing imaging methods as ESEM, TEM, and SEM.

## 4. Conclusions

Synthetic ettringite was chosen as model system to investigate the impact of different levels of low-pressure treatment (4 mbar, 60 µbar, 2.8 nbar) on its chemical composition, crystal structure, and morphology during characterization measurements and the drying process. It was shown that the level as well as the duration of exposure to low pressure is of importance, as a lower pressure dehydrates ettringite faster. The bound water content in treated ettringite, derived from thermogravimetric analysis (TGA), shows at 4 mbar, a linear decrease of 14% over 72 h and at 60 µbar, a dehydration of 67% within 48 h, following an exponential decrementary. These findings are supported by the retrogression of the characteristic X-ray diffraction (XRD) pattern of ettringite and by the change of the cell parameters of ettringite, derived by selected area electron diffraction (SAED) as well as Pawley fits of the XRD data. SAED additionally revealed that despite phase conversion of ettringite due to dehydration, no change to the underlying crystal symmetry occurs. Raman measurements confirm that water loss comes mostly from bound water and not from adsorbed water. Our findings suggest that morphological investigation on a microscopic scale should only be conducted (if possible) using noninvasive characterization techniques requiring a low vacuum, such as environmental scanning electron microscopy (ESEM) or liquid cell transmission electron microscopy (LCTEM) [39]. The results of this manuscript might help future work, for example, in evaluating the crystal growth of early hydration products in cementitious suspensions.

## Data Availability

Data supporting reported results can be acquired per request to the corresponding author.

## Supplementary Materials

The following are available online at https://www.mdpi.com/article/10.3390/ma14112720/s1, Figure S1. Crystal structure of ettringite following Hartman et al. [1]; (a) part of a single column in [$10\bar{1}0$] projection and (b) schematic build-up in the [0001] projection. Figure S2. Water content of synthetic ettringite derived from (a) balance and (b) TGA after treatment at various level of low pressure (4 mbar: black; 60 µbar: green) for different durations (up to 72 h) calculated on the assumption that during heat treatment, water is the sole decomposition product; (c) mass increase of treated ettringite (24 h at 60 µbar) over time due to relative humidity in ambient air. Figure S3. Mass loss of synthetic ettringite measured (a) by balance and (b) through TGA after treatment at two different levels of low pressure (4 mbar: black; 60 µbar: green) for different durations (up to 72 h); (c) combined assessment of mass loss of synthetic ettringite by balance and TGA after treatment at two different levels of low pressure (4 mbar: black; 60 µbar: green) for various durations (up to 72 h). Figure S4. TGA data plotted as mass normalized weight loss (dΔm·(dt)^−1^) per hour against the temperature of synthetic ettringite with a heating rate of 5 °C/min after treatment at two different levels of low pressure (a) 4 mbar and (b) 60 µbar for various durations (up to 72 h); full time frame. Figure S5. TGA data plotted as mass normalized weight loss (dΔm·(dt)^−1^) per hour against the temperature of synthetic ettringite with a heating rate of 5 °C/min after treatment at three different levels of low pressure; (a) steepness of first peak; (b) comparison of 4 mbar, 60 µbar, and 2.8 nbar. Figure S6. Retrogression of the characteristic diffraction pattern of synthetic ettringite after treatment at two different levels of pressures (4 mbar and 60 µbar) for various durations (up to 72 h); Bravais–Miller indices are given accordingly to a trigonal symmetry in a hexagonal cell; full time frame. Figure S7. Correlation between the retrogression derived by Pawley fit of ettringite’s (a) cell parameter a, (b) cell parameter c, (c) cell volume V after treatment at three different levels of low pressure (4 mbar: black; 60 µbar: red; 2.8 nbar: green) for various durations (up to 72 h), (d) Raman spectroscopy, and (e) TGA of pristine synthetic ettringite and treated ettringite after treatment at two different levels of low pressure (4 mbar for 72 h and 60 µbar for 48 h); TGA data plotted as mass normalized time derived weight loss (dΔm·(dt)^−1^) per hour against the reference temperature of synthetic ettringite with a heating rate of 5 °C/min. Figure S8. ESEM; ESEM micrographs of synthetic ettringite after treatment at two different levels of pressure (a) 1 mbar for 2 min and (b) 35.3 nbar for 2 min. Table S1. Mass loss of synthetic ettringite measured by balance after treatment at different levels of reduced pressure for various durations; m_0_ (t) = 5.00 g; m_0,H_2_O_ (t) = 2.30 g; device specific deviation: ± 0.1 mg. Table S2. Mass loss of synthetic ettringite calculated after TGA after treatment at different levels of low pressure for various durations; m_0_ (t) = 51.10 mg ± 2.39 mg; m_0,H_2_O_ (t) = 23.47 mg ± 1.10 mg; device specific deviation: ±0.005% (min. 1 µg). Table S3. Equations of the decay of the first decomposition peak during TGA measurements after treatment at different levels of low pressure (4 mbar and 60 µbar). Table S4. Vibration bands of pristine ettringite, of ettringite treated for 72 h at 4 mbar, of ettringite treated for 48 h at 60 µbar, and of ettringite exposed to the reduced pressure in the antechamber of the SEM decreasing to 2.8 nbar within 20 min. Table S5. Data for Figure 8; retrogression of the cell of synthetic ettringite after treatment at two different levels of low pressure (4.0 mbar and 60 µbar) for various durations (up to 72 h); R_wp_, R_exp_ and GOF derived by Pawley fit [4,5] [19,40]. Table S6. Data for Figure 8; retrogression of the cell of synthetic ettringite; sample exposed to the reduced pressure in the antechamber of the SEM decreasing to 2.8 nbar within 20 min; R_wp_, R_exp_ and GOF derived by Pawley fit [4,5].
