# Supplementary material for: Influence of Low-Pressure Treatment on the Morphological and Compositional Stability of Microscopic Ettringite
Source: Materials (Basel). 2021 May 21;14(11):2720. doi: 10.3390/ma14112720 (PMC8196742; doi:10.3390/ma14112720)
Supplement: Supplementary file 1 [file materials-14-02720-s001.zip › materials-1175823-supplementary.pdf]

materials

MDPI

Supporting Information

Influence of Low-Pressure Treatment on the Morphological and Compositional Stability of Microscopic Ettringite

Patrick A. Kißling [1], Franziska Lübkemann [1], Tabea von Bronk [2], Dario Cotardo [2], Lei Lei [3], Armin Feldhoff [1], Ludger Lohaus [2], Michael Haist [2] and Nadja C. Bigall [1,*]

[1] Institute of Physical Chemistry and Electrochemistry, Leibniz Universität Hannover, 30167 Hanover, Germany; patrick.kissling@pci.uni-hannover.de (P.A.K.); franziska.luebkemann@pci.uni-hannover.de (F.L.); armin.feldhoff@pci.uni-hannover.de (A.F.)

[2] Institute of Building Materials Science, Leibniz Universität Hannover, 30167 Hanover, Germany; t.von-bronk@baustoff.uni-hannover.de (T.v.B.); d.cotardo@baustoff.uni-hannover.de (D.C.); lohaus@baustoff.uni-hannover.de (L.L.); haist@baustoff.uni-hannover.de (M.H.)

[3] Chair for Construction Chemistry, Technische Universität München, 85747 Munich, Germany; lei.lei@bauchemie.ch.tum.de

* Correspondence: nadja.bigall@pci.uni-hannover.de; Tel.; +49-511-762-14439

Citation: Kißling, P.A.; Lübkemann, F.; von Bronk, T.; Cotardo, D.; Lei, L.; Feldhoff, A.; Lohaus, L.; Haist, M.; Bigall, N.C. Influence of Low-Pressure Treatment on the Morphological and Compositional Stability of Microscopic Ettringite. *Materials* **2021**, *14*, 2720. https://doi.org/10.3390/ma14112720

Academic Editors: Jose Antonio Alonso and Daniela Kovacheva

Received: 24 March 2021
Accepted: 17 May 2021
Published: 21 May 2021

Publisher's Note: MDPI stays neutral with regard to jurisdictional claims in published maps and institutional affiliations.

Copyright: © 2021 by the authors. Licensee MDPI, Basel, Switzerland. This article is an open access article distributed under the terms and conditions of the Creative Commons Attribution (CC BY) license (http://creativecommons.org/licenses/by/4.0/).

Abstract: The impact of low-pressure treatment on the crystal structure, morphology, and chemical composition of ettringite, due to their major importance with respect to processability (i.a., drying conditions) and to the analysis of ettringite-containing samples, is examined utilizing X-ray diffraction, thermogravimetric analysis, Raman spectroscopy, and environmental scanning electron microscopy. Synthetic ettringite was treated for various durations (5 min up to 72 h) and at two different levels of low-pressure (4.0 mbar and 60 µbar). Evaluation showed a correlation between the procedural parameters (time and pressure), the chemical composition, and the morphology of ettringite. The experiments reveal that, when exposed to 4 mbar pressure, nearly no changes occur in the ettringite's morphology, whereas the crystals undergo swelling and slight deformations at very low pressures (60 µbar and 35.3 nbar), which is attributed to the loss of bound water and the partial transformation from ettringite to quicklime, anhydrite, and calcium aluminate. Furthermore, the strongly dehydrated ettringite shows the same morphology.

Keywords: morphology; chemical composition; ettringite; low-pressure; Pawley fit

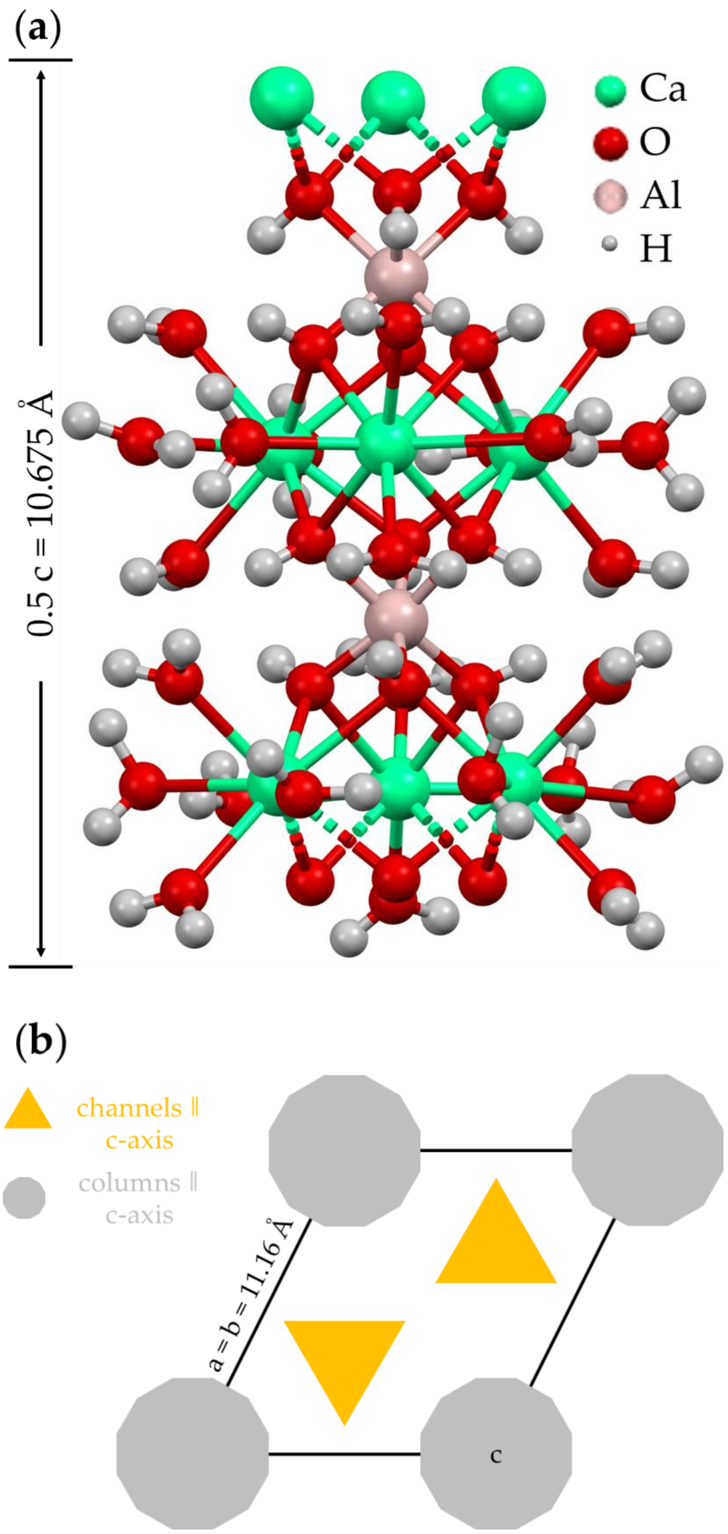

Figure S1. Crystal structure of ettringite following Hartman et al. [1]; (**a**) part of a single column in the [10$\bar{1}$0] projection and (**b**) schematic buildup in the [0001] projection.

By simple mass measurements using a balance, the loss of water was investigated gravimetrically after treatment with low pressure for different durations (see **Figure S2a,b** and **Tables S1** and S2). A slightly reduced pressure of 4.0 mbar manages to extract only 6.4 wt% of bound water after 72 h. A similar degree of extraction (6.7 wt % of bound water) is reached after 2 h at a pressure of 60 µbar. After 24 h the low-pressure treatment at 60 µbar leads to a mass loss of 30.2 wt% of bound water, which then stays nearly constant for at least 24 h.

A similar curve can be derived from the maximal difference in mass over the whole decomposition range of TGA measurements (see **Figures S2 and S3** and **Table S2**). The obtained maximum discrepancy in mass loss of 10% between balance and TGA measurements is most probably induced by the loss of small grains during vacuum application: less loss with less vacuum, after 24 h at 60 µbar around 1 %. The main factor is the time needed to transfer the sample from the freeze dryer to the TGA device to complete the weigh-in and to start the measurement, as the relative humidity in the air leads to rehydration of the activated ettringite over time (see **Figure S2c**). Measuring the mass loss by balance gives a good first impression of the extracted bound water.

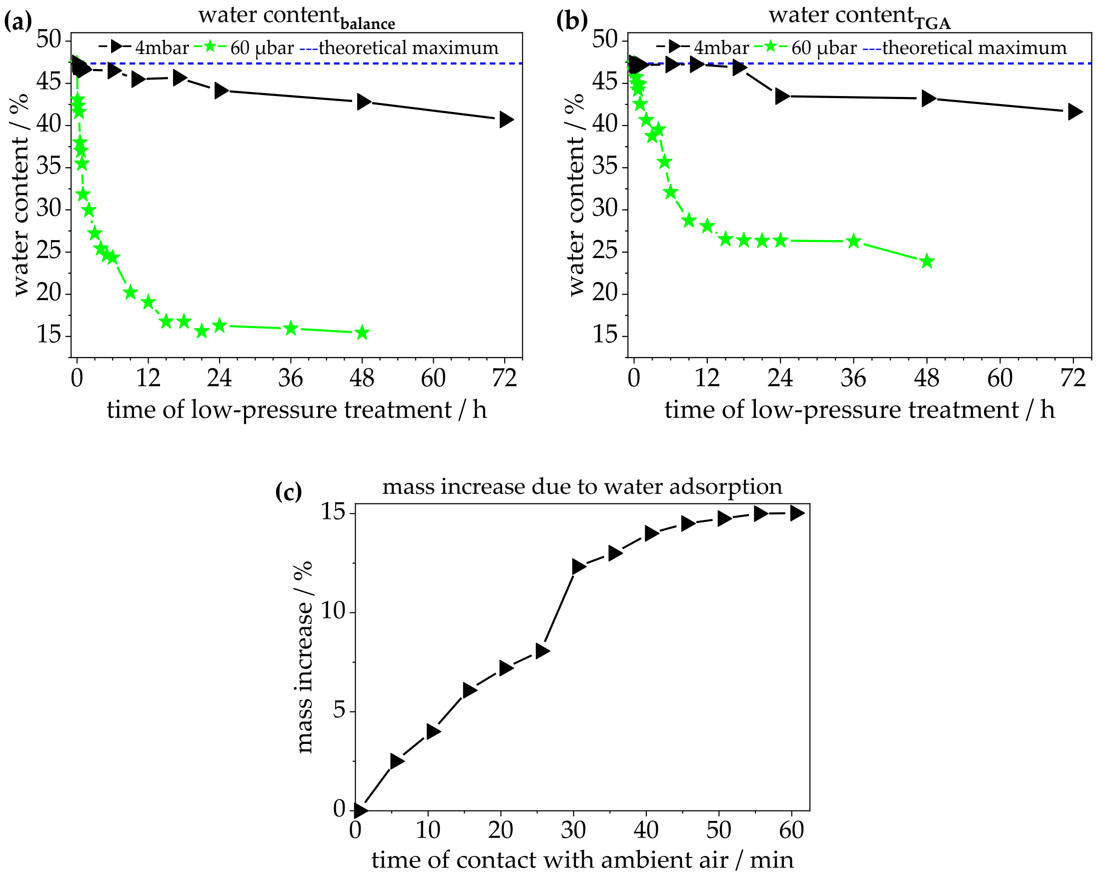

Figure S2. Water content of synthetic ettringite derived from (**a**) balance and (**b**) TGA after treatment at various level of low pressure (4 mbar: black; 60 µbar: green) for different durations (up to 72 h); calculated on the assumption that during heat treatment, water is the sole decomposition product; (**c**) mass increase of treated ettringite (24 h at 60 µbar) over time due to relative humidity in ambient air.

The extent of mass loss decreases according to the higher extraction of bound water induced by a higher level and longer time of exposure to low pressure, so less water is still inside the crystal structure. The higher the extraction rate of bound water, the lower the mass loss induced by decomposition within TGA. Therefore, the course of the mass loss must be inversely proportional to that measured by the balance, which is depicted in

Figure S3. At a slightly reduced pressure of 4 mbar, the mass loss (Δm) stays around 100.34 % ± 0.49 % within the first 17 h. Afterwards, the mass loss is underestimated at 94.94 % ± 0.49 %, so in total, there is a slight underestimation in the range of device error of 99.10 % ± 2.33 % (see **Figure S3**). This statement is not valid for the exposure to lower pressure (60 µbar). In the first 4 h, the desorbed amount is exaggerated by 3.53 % ± 1.40 %, and for the remaining 44 h, the underestimation is at 85.12 % ± 8.61 %, so in total, there is an underestimation of 5.67 % ± 11.08 %.

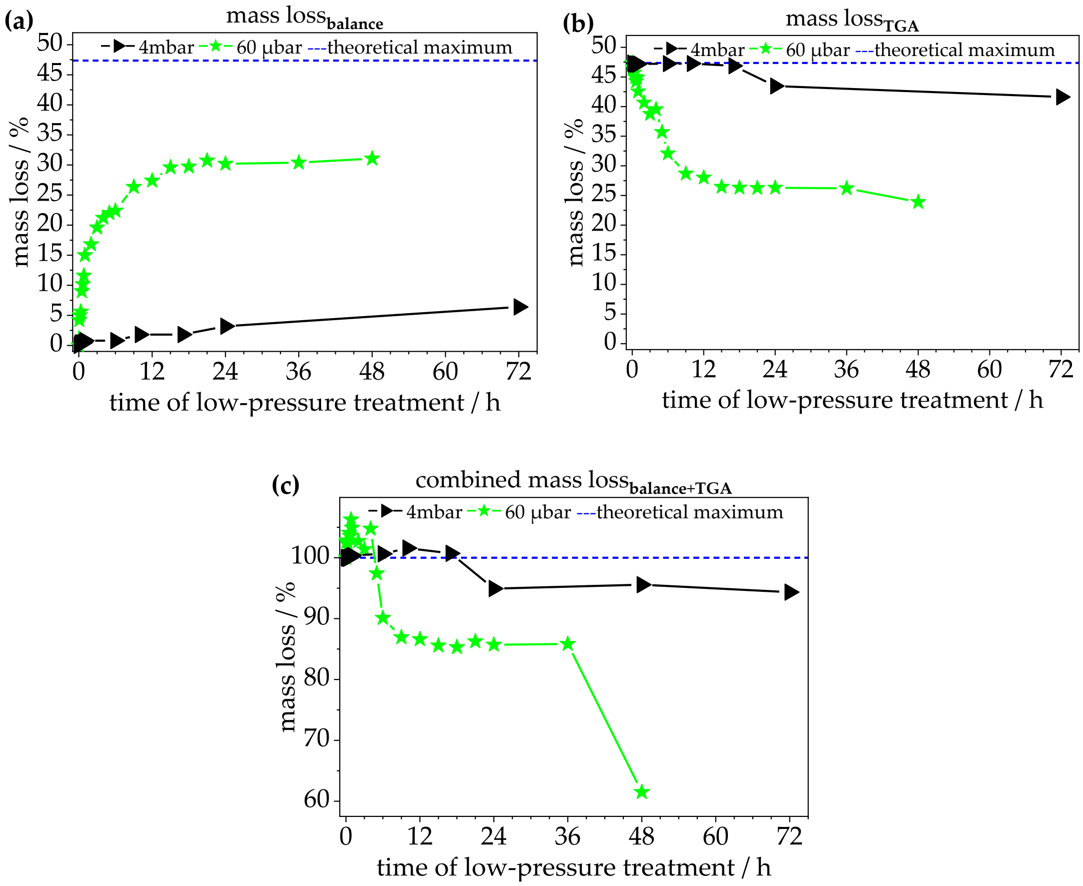

Figure S3. Mass loss of synthetic ettringite measured (**a**) by balance and (**b**) through TGA after treatment at two different levels of low pressure (4 mbar: black; 60 µbar: green) for different durations (up to 72 h); (**c**) combined assessment of mass loss of synthetic ettringite by balance and TGA after treatment at two different levels of low pressure (4 mbar: black; 60 µbar: green) for various durations (up to 72 h).

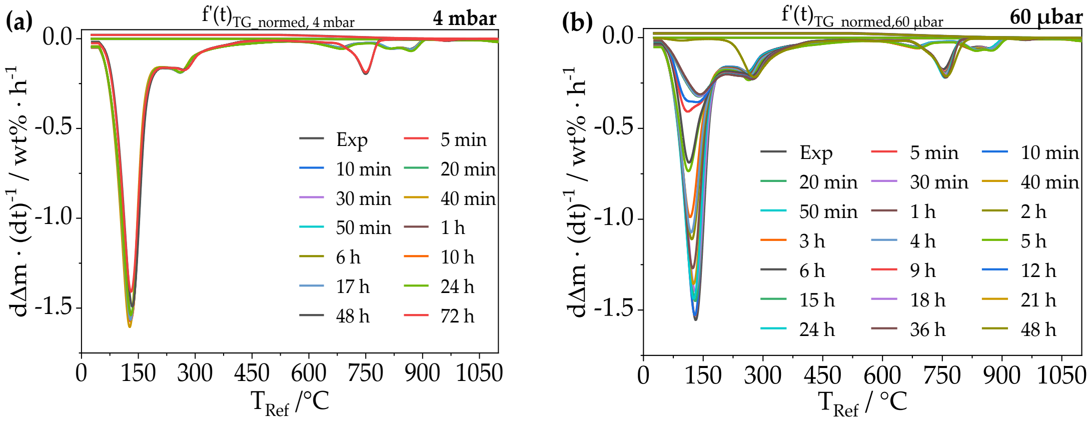

Figure S4. TGA data plotted as mass normalized time derived weight loss (dΔm · $(dt)^{-1}$) per hour against the reference temperature of synthetic ettringite with a heating rate of 5 °C/min after treatment at two different levels of low pressure, (**a**) 4 mbar and (**b**) 60 µbar, for various durations (up to 72 h); full-time frame.

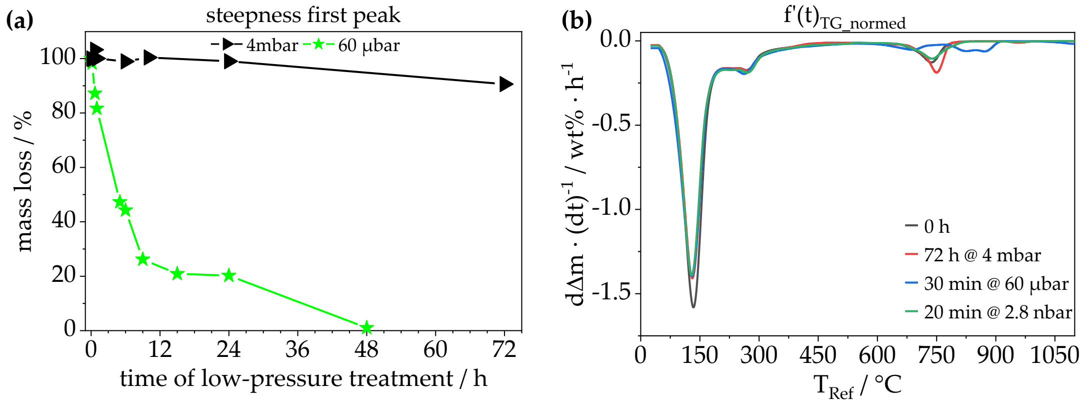

Figure S5. TGA data plotted as mass normalized time derived weight loss (dΔm · $(dt)^{-1}$) per hour against the reference temperature of synthetic ettringite with a heating rate of 5 °C/min after treatment at different levels of low pressure; (**a**) steepness of the first peak; (**b**) comparison of 4 mbar, 60 µbar, and 2.8 nbar.

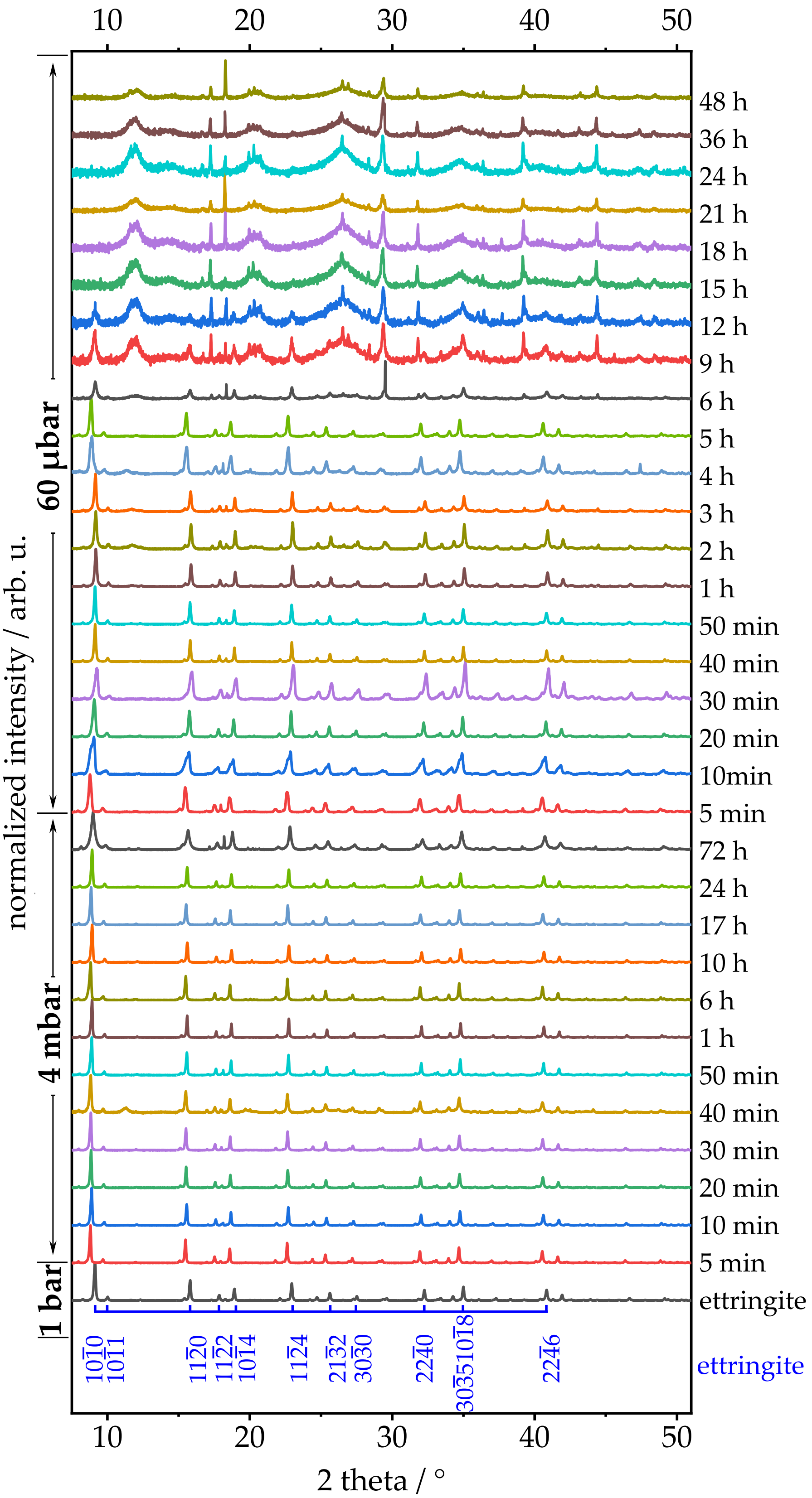

Figure S6. Retrogression of the characteristic diffraction pattern of synthetic ettringite after treatment at two different levels of pressures (4 mbar and 60 µbar) for various durations (up to 72 h); Bravais–Miller indices are given according to a trigonal symmetry in a hexagonal cell; full-time frame.

The comparison of retrogression derived from Pawley fit of XRD data with the results of Raman spectroscopy and TGA of samples treated for 72 h at 4 mbar and 48 h at 60 µbar shows correlation, (see **Figure S7**). Raman and TGA depict that at 60 µbar, the bound water content is at its minimum. At this point, the cell parameters are also at their lowest values. In case of the sample treated at 4 mbar for 72 h, nearly no change in the cell parameters was detected, which is in good correlation with the TGA and Raman measurements. In both cases, no significant change to the bound water content (Raman at 3500 cm-1 and TGA at 150 °C) was found.

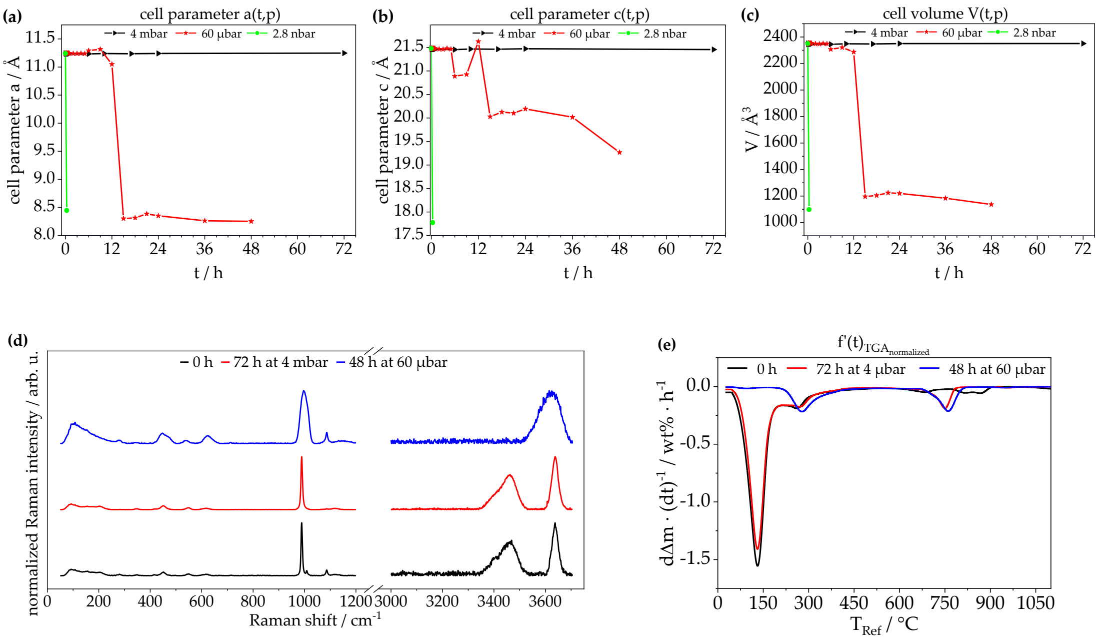

Figure S7. Correlation between the retrogression derived by Pawley fit of ettringite's (**a**) cell parameter **a**, (**b**) cell parameter **c**, and (**c**) cell volume **V** after treatment at three different levels of low pressure (4 mbar: black; 60 µbar: red; 2.8 nbar: green) for various durations (up to 72 h), (**d**) Raman spectroscopy, and (**e**) TGA of pristine synthetic ettringite and treated ettringite; both (**d+e**) after treatment at two different levels of low pressure (4 mbar for 72 h and 60 µbar for 48 h); TGA data plotted as mass normalized time derived weight loss (dΔm · (dt)-1) per hour against the reference temperature of synthetic ettringite with a heating rate of 5 °C/min.

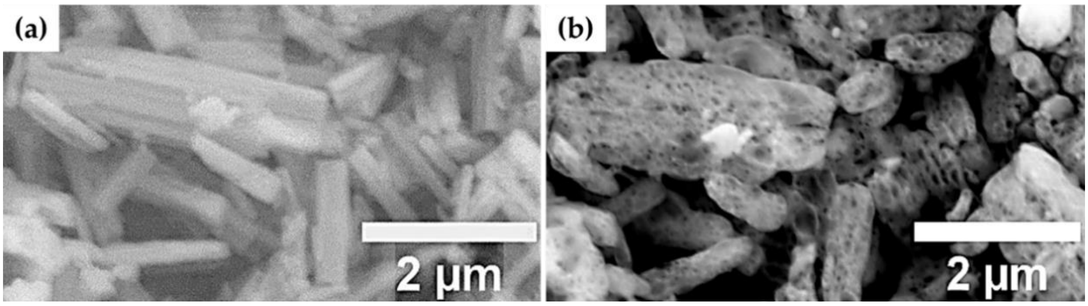

Figure S8. ESEM; ESEM micrographs of synthetic ettringite after treatment at two different levels of pressure, (**a**) 1 mbar for 2 min and (**b**) 35.3 nbar for 2 min.

Table S1. Mass loss of synthetic ettringite measured by balance after treatment at different levels of reduced pressure for various durations; $m_0(t) = 5.00$ g; $m_{0, H_2O}(t) = 2.30$ g; device-specific deviation: ± 0.1 mg.

4.0 mbar						60 µbar				
**m(t) / g**	**Δm(t) / g**	**Δm(t) / %**	**$\Delta m_{H_2O}(t)$ / %**	**$\Delta n_{H_2O}(t)$ / mmol**	**t / h**	**m(t) / g**	**Δm(t) / g**	**Δm(t) / %**	**$\Delta m_{H_2O}(t)$ / %**	**$\Delta n_{H_2O}(t)$ / mmol**
5.00	0.00	0.00	0.00	0.00	0	5.00	0.00	0.00	0.00	0.00
5.00	0.01	0.20	0.43	0.00	0.083	4.79	0.21	4.20	9.14	0.36
4.99	0.02	0.40	0.87	0.03	0.17	4.76	0.25	4.99	10.86	0.43
4.99	0.02	0.40	0.87	0.03	0.33	4.72	0.28	5.60	12.19	0.49
4.98	0.02	0.40	0.87	0.03	0.50	4.55	0.45	9.00	19.59	0.78
4.98	0.02	0.40	0.87	0.03	0.67	4.50	0.51	10.18	22.16	0.88
4.97	0.03	0.60	1.31	0.05	0.83	4.42	0.58	11.60	25.25	1.01
4.97	0.04	0.80	1.74	0.07	1	4.25	0.75	15.00	32.66	1.30
					2	4.16	0.84	16.80	36.58	1.46
					3	4.02	0.98	19.60	42.67	1.70
					4	3.94	1.06	21.20	46.16	1.84
					5	3.90	1.10	22.00	47.90	1.91
4.96	0.04	0.80	1.74	0.07	6	3.88	1.12	22.40	48.77	1.94
					9	3.69	1.32	26.35	57.36	2.29
4.91	0.09	1.80	3.92	0.16	10					
					12	3.63	1.37	27.40	59.65	2.38
					15	3.52	1.48	29.60	64.44	2.57
4.92	0.09	1.80	3.91	0.16	17					
					18	3.52	1.49	29.74	64.75	2.58
					21	3.47	1.54	30.74	66.92	2.67
4.85	0.16	3.19	6.95	0.28	24	3.49	1.51	30.20	65.75	2.62
					36	3.48	1.52	30.40	66.19	2.64
					48	3.44	1.55	31.06	67.63	2.69
4.68	0.32	6.40	13.93	0.56	72					

Table S2. Mass loss of synthetic ettringite calculated after TGA after treatment at different levels of low pressure for various durations; m_0 (t) = 51.10 mg ± 2.39 mg; m_{0, H_2O} (t) = 23.47 mg ± 1.10 mg; device-specific deviation: ± 0.005 % (min. 1 µg).

4.0 mbar						60 µbar				
m(t) / mg	Δm(t) / mg	Δm(t) / %	$\Delta m_{H_2O}(t)$ / %	$\Delta n_{H_2O}(t)$ / µmol	t / h	m(t) / mg	Δm(t) / mg	Δm(t) / %	$\Delta m_{H_2O}(t)$ / %	$\Delta n_{H_2O}(t)$ / µmol
28.72	25.84	47.35	103.09	44.81	0	28.72	25.84	47.35	103.09	44.81
26.48	23.65	47.18	102.73	41.03	0.083	27.31	23.85	46.63	101.51	41.38
26.48	23.65	47.18	102.73	41.03	0.17	26.56	22.97	46.38	100.97	39.85
28.53	25.42	47.12	102.59	44.10	0.33	28.14	23.70	45.71	99.52	41.10
28.32	25.30	47.18	102.72	43.89	0.50	29.67	24.27	44.99	97.94	42.09
24.59	21.96	47.17	102.70	38.09	0.67	28.27	22.45	44.26	96.35	38.94
29.31	26.14	47.14	102.64	45.34	0.83	26.53	21.58	44.86	97.66	37.43
27.97	24.99	47.19	102.73	43.36	1	27.67	20.49	42.55	92.63	35.54
					2	30.96	21.20	40.65	88.49	36.78
					3	28.42	17.98	38.75	84.37	31.20
					4	30.46	19.91	39.53	86.06	34.54
					5	32.51	18.04	35.69	77.70	31.29
27.73	24.82	47.23	102.83	43.05	6	32.44	15.33	32.09	69.87	26.59
					9	36.34	14.61	28.68	62.44	25.35
26.86	24.05	47.25	102.86	41.73	10					
					12	37.92	14.76	28.01	60.99	25.60
					15	35.59	12.80	26.46	57.60	22.21
26.08	23.01	46.87	102.05	39.91	17					
					18	37.59	13.43	26.33	57.31	23.30
					21	37.57	13.38	26.26	57.17	23.20
30.28	23.29	43.47	94.65	40.40	24	37.15	13.26	26.30	57.27	23.00
					36	39.58	14.06	26.21	57.06	24.39
					48	40.06	12.58	23.90	52.03	21.82
30.13	21.48	41.62	90.61	37.26	72					

Table S3. Equations of the decay of the first decomposition peak during TGA measurements after treatment at different levels of low pressure (4 mbar and 60 µbar).

pressure	function	value		
4 mbar	$y = a + b \cdot x$	a = 100.66228 ± 0.4453	b = -0.11878 ± 0.01808	
60 µbar	$y = a - b \cdot c^x$	a = 18.59904 ± 1.19564	b = -81.34841 ± 1.421	c = 0.80861 ± 0.00983

Table S4. Vibration bands of pristine ettringite, of ettringite treated for 72 h at 4 mbar, of ettringite treated for 48 h at 60 μbar, and of ettringite exposed to the reduced pressure in the antechamber of the SEM (decreasing to 2.8 nbar within 20 min).

		Literature [2,3] / cm^{-1}	Ettringite 0 h / cm^{-1}	72 h at 4 mbar / cm^{-1}	48 h at 60 μbar / cm^{-1}	20 min at 2.8 nbar / cm^{-1}
100 cm^{-1} to 12000 cm^{-1}	υ_2 [SO_4]	452.1 ± 15.8	451.5 ± 28.5	452.5 ± 36.5	455.8 ± 49.8	454.3 ± 29.8
	'[$Al(OH)_6$]'	548.7 ± 10.2	536.5 ± 11.5	548.8 ± 25.8	543.8 ± 31.3	548.0 ± 20.5
	υ_4 [SO_4]	610.2 ± 22.2	612.0 ± 26.0	618.0 ± 29.5	625.3 ± 47.3	620.0 ± 20.0
	υ_1 [SO_4]	988.5 ± 4.7	988.5 ± 30.5	990.3 ± 32.3	996.8 ± 45.8	986.8 ± 20.8
	υ_3 [SO_4]	1120.0 ± 25.6	1086.0 ± 20.0	1088.5 ± 29.5	1086.5 ± 28.0	- - - - -
3000 cm^{-1} to 3700 cm^{-1}	[H_2O] stretching	3289.2 ± 199.4	3444.8 ± 102.8	3434.8 ± 96.8	- - - -	3440.8 ± 90.3
	[H_2O] stretching	3404.7 ± 75.6				
	[H_2O] stretching	3464.2 ± 107.8				
	[H_2O] stretching	3517.4 ± 107.8				
	[OH] stretching	3638.0 ± 18.5	3644.0 ± 42.0	3640.0 ± 46.0	3606.3 ± 98.8	3644.4 ± 42.4

Table S5. Data for **Figure 8**; retrogression of the cell parameter of synthetic ettringite after treatment at two different levels of low pressure (4.0 mbar and 60 μbar) for various durations (up to 72 h); R_{wp}, R_{exp} and GOF derived by Pawley fit [4,5].

4.0 mbar							60 μbar					
a(t) / Å	c(t) / Å	V(t) / Å^3	R_{wp}	R_{exp}	GOF	t / h	a(t) / Å	c(t) / Å	V(t) / Å^3	R_{wp}	R_{exp}	GOF
11.24	21.48	2351	2.32	8.06	3.48	0	11.24	21.48	2351	2.32	8.06	3.48
11.23	21.46	2345	1.53	15.5	10.1	0.083	11.24	21.46	2346	1.74	16.4	9.43
11.24	21.47	2348	1.54	16.5	10.7	0.17	11.25	21.49	2355	1.79	13.8	7.71
11.24	21.47	2349	1.57	15.4	9.83	0.33	11.25	21.48	2352	1.58	15.5	9.76
11.24	21.46	2347	1.54	15.9	10.3	0.50	11.24	21.48	2352	1.83	14.3	7.83
11.24	21.46	2348	1.59	11.1	6.96	0.67	11.25	21.48	2353	1.65	13.2	7.97
11.24	21.46	2347	1.51	17.3	11.4	0.83	11.25	21.48	2353	1.67	10.0	5.99
11.24	21.46	2348	1.55	17.4	11.3	1	11.24	21.48	2351	1.75	8.05	4.61
						2	11.24	21.47	2349	1.75	11.4	6.49
						3	11.24	21.46	2348	1.74	9.01	5.19
						4	11.24	21.48	2351	1.53	7.99	5.22
						5	11.24	21.47	2348	1.48	16.8	11.4
11.23	21.45	2345	1.63	19.5	11.9	6	11.29	20.89	2307	1.57	2.98	1.90
						9	11.32	20.93	2321	1.56	2.95	1.88
11.24	21.47	2349	1.57	17.1	10.9	10						
						12	11.05	21.63	2287	1.58	3.89	2.47
						15	8.30	20.03	1195	1.58	2.05	1.30
11.24	21.46	2347	1.55	14.8	9.58	17						
						18	8.31	20.13	1205	1.57	4.15	2.64
						21	8.39	20.10	1224	1.63	2.63	1.62
11.24	21.47	2350	1.56	16.3	10.4	24	8.35	20.19	1220	1.57	2.41	1.53
						36	8.26	20.02	1183	1.59	2.42	1.52
						48	8.25	19.27	1136	1.22	1.88	1.54
11.25	21.46	2351	1.63	9.47	5.83	72						

Table S6. Data for **Figure 8**; retrogression of the cell of synthetic ettringite; sample exposed to the reduced pressure in the antechamber of the SEM decreasing to 2.8 nbar within 20 min; R_{wp}, R_{exp} and GOF derived by Pawley fit [4,5].

		2.8 nbar					
**a(t) /Å**	**c(t) / Å**	**V(t) / Å³**	**R_{wp}**	**R_{exp}**	**GOF**	**t / h**	
11.24	21.48	2351	2.32	7.85	3.54	0	
8.45	17.77	1098	0.73	3.99	5.48	0.33	

References

1. Hartman, M.R.; Berliner, R. Investigation of the structure of ettringite by time-of-flight neutron powder diffraction techniques. *Cem. Concr. Res.* **2006**, *36*, 364–370, doi:10.1016/j.cemconres.2005.08.004.
2. Renaudin, G.; Filinchuk, Y.; Neubauer, J.; Goetz-Neunhoeffer, F. A comparative structural study of wet and dried ettringite. *Cem. Concr. Res.* **2010**, *40*, 370–375, doi:10.1016/j.cemconres.2009.11.002.
3. Renaudin, G.; Segni, R.; Mentel, D.; Nedelec, J.M.; Leroux, F.; Taviot-Gueho, C. A Raman study of the sulfated cement hydrates: Ettringite and monosulfoaluminate. *J. Adv. Concr. Technol.* **2007**, *5*, 299–312, doi:10.3151/jact.5.299.
4. Pawley, G.S. Unit-cell refinement from powder diffraction scans. *J. Appl. Crystallogr.* **1981**, *14*, 357–361, doi:10.1107/S0021889881009618.
5. Toby, B.H. R factors in Rietveld analysis: How good is good enough? *Powder Diffr.* **2006**, *21*, 67–70, doi:10.1154/1.2179804.
